# Associated factors of presenteeism in a random sample of Portuguese nurses: a cross-sectional study

**DOI:** 10.1192/j.eurpsy.2023.550

**Published:** 2023-07-19

**Authors:** J. Borges, C. Laranjeira

**Affiliations:** ^1^Pediatric Ward, Hospital of Figueira da Foz, Figueira de Foz; ^2^ School of Health Sciences; ^3^ciTechCare, Polytechnic of Leiria, Leiria, Portugal

## Abstract

**Introduction:**

Presenteeism is increasingly being seen as a threat to employee efficiency. Nursing professionals are exposed to occupational hazards that may compromise physical and mental health, interfere with the quality of life of the worker and the quality of care provided to the patient and cause illness and generate costs for the institutions.

**Objectives:**

This study aimed to evaluate which are the personal (age, sex, educational qualifications), professional (organization, seniority in the organization, seniority in function, employment relationship, work regime and professional category) and health variables, that best relate to sickness presenteeism.

**Methods:**

A quantitative cross-sectional study was conducted. The sample is probabilistic and the universe of this study included all nurses who work in Portuguese health institutions (whether they are of a public, private or public-private nature). Inclusion criteria were nurses with clinical activity and/or management in institutions in the aforementioned modalities, and nurses in teaching activity in higher education institutions were excluded from this study, on an exclusive basis. The final sample was composed of 424 nurses.

**Results:**

Most of the nurses who answered the e-survey are female (86.8%), aged 40 years or over (59.9%), who live in the northern region of the country (31.4%), who work in public organizations (85.5%), in differentiated health care units (52.6%) and with an employment contract in public functions (63.7%). More than half (53.3%) of participants are specialist nurses and 72.4% of respondents did not have a previous disease condition. The variables that best correlated with presentism were the female gender, the organization where you work (public), and the presence of a previous disease condition.

**Conclusions:**

The results presented may constitute a challenge for change for policymakers, managers and health professionals, in the sense of addressing presenteeism with the creation of occupational health programs directed to the needs of nurses. Other measures include flexible working hours, work policies that promote staff retention, and a strong commitment to the training and qualification of nurses capable of ensuring better performance and involvement in the organizational culture.

**Disclosure of Interest:**

None Declared

